# Non-Invasive Methodology to Estimate Polyphenol Content in Extra Virgin Olive Oil Based on Stepwise Multilinear Regression

**DOI:** 10.3390/s18040975

**Published:** 2018-03-25

**Authors:** Diego Manuel Martínez Gila, Pablo Cano Marchal, Juan Gómez Ortega, Javier Gámez García

**Affiliations:** Robotics, Automation and Computer Vision Group, University of Jaen, Campus Las Lagunillas s/n, ES-23071 Jaen, Spain; pcano@ujaen.es (P.C.M.); juango@ujaen.es (J.G.O.); jggarcia@ujaen.es (J.G.G.)

**Keywords:** olive oil production process, near infrared spectrum, polyphenol content, feature extraction

## Abstract

Normally the olive oil quality is assessed by chemical analysis according to international standards. These norms define chemical and organoleptic markers, and depending on the markers, the olive oil can be labelled as lampante, virgin, or extra virgin olive oil (EVOO), the last being an indicator of top quality. The polyphenol content is related to EVOO organoleptic features, and different scientific works have studied the positive influence that these compounds have on human health. The works carried out in this paper are focused on studying relations between the polyphenol content in olive oil samples and its spectral response in the near infrared spectra. In this context, several acquisition parameters have been assessed to optimize the measurement process within the virgin olive oil production process. The best regression model reached a mean error value of 156.14 mg/kg in leave one out cross validation, and the higher regression coefficient was 0.81 through holdout validation.

## 1. Introduction

The CE61/2011 regulation and the International Olive Council characterize the commercial olive oil quality according to several parameters related to the deterioration state of the virgin olive oil (VOO). These parameters include the free acidity and several others that are related to the oxidation state of the VOO, such as the index of peroxides and the K_232_, K_270_, and ∆K absorption indexes [1]. Other minor compounds, such as phenols, are related to the quality and sensory attributes of olive oil, and play an important role in the positive health properties of the VOO [2]. Furthermore, the consumer choice can be influenced by these features, and it can be useful for competitive strategies and marketing.

The virgin olive oil production process starts in the reception yard where the farmers put their olive fruit into the reception hopper. The olive fruits are conveyed by belts to the industrial washing machine, where stones and mud are removed from the olive batches. Then, they are weighted and stored in huge hoppers before the milling process. In this process, olives are crushed and turned into olive paste. The next step is the thermomixer, where the olive paste is constantly heated and stirred during 90 min. After that, the olive paste is pumped to the horizontal centrifuge machine where the liquid oil is separated from the olive paste. At the end, it is cleaned and stored into the cellar. A more detailed explanation of the process can be found in [3].

The olive oil compounds depend fundamentally on physicochemical features of the fruit, although they are modulated according to the technological process variables. Through the years, different works have examined the influence of technological operations of olive processing on oil yields and quality [4,5], and that knowledge can be used to implement the Process Analytical Technology (PAT) concept into the olive oil sector. That concept was introduced in 2004 [6], and originally, it was focused on the pharmaceutical industry, having been introduced in the food industry recently [7]. PAT employs non-invasive sensors working inline and online, such as Near Infrared (NIR), biosensors, and electronic noses, in order to automatically acquire information from the process. According to this information it learns, the process (through chemometric procedures) can be useful to detect and correct deviations [8]. It has four main components [9]: studying the relationship between product features and process factors; exploring how the process dynamic can affect the sampling procedure; choosing the analytical instruments for inline or online process stream measurements, and employing algorithms to analyze multivariate data. The previous works presented in Section 2 are based on those points.

The goals of this work are focused on the second and fourth points of the PAT concept. According to this concept, two aims were defined: the first was to find correlation model between the olive oil NIR spectra and high concentrations of polyphenols by means of employing algorithms based on feature extraction approaches; the second goal was to study the influence in the prediction models of several acquisition parameters, such as number of acquisitions per sample, and light source power is analyzed in the context of the application of an online NIR sensor within the Virgin Olive Oil Production Process (VOOPP).

This paper has been organized as follows: Section 2 presents the problem description and describes previous works; Section 3 shows the methodology, the hardware that has been used, and the mathematical resources employed to get the results; Section 4 explains the outcomes; Section 5 presents the conclusions.

## 2. Problem Description and Earlier Works

Nowadays, the master miller is the person in the factory who adjusts the process variables based on his own expertise, because the produced olive oil is analyzed in laboratories far from the factory. This scheme is prone to lack of objectivity, and sometimes the decisions cannot be based on the results because of the delay. Furthermore, the methods employed to assess the olive oil quality are invasive, time consuming, expensive, and require qualified staff.

Different works have evaluated the use of non-invasive technologies, such as near infrared spectrometers, computer vision, or electronic noses and tongues, to monitor the product qualities at all stages of the production process [10,11,12,13,14,15]. Particularly, the application of the near infrared (NIR) technology on the olive oil industry has been reported in [16]. In that case, the authors reviewed the literature related to the use of NIR spectrometry in the olive oil industry over olive fruits, slurries, and olive oils. Furthermore, an interesting review was done in [17], where the authors divided the main applications of olive oil NIR spectroscopy into adulteration detection, prediction of geographical origin, quality parameter determination, online process monitoring, and oxidative stability studies.

Particularly, the use of NIR spectrum for the determination of minor components of VOO has been reported in several studies instead of an optical spectrum. This is because the X–H molecular bonds of these minor compounds vibrate in the near infrared frequencies when they are excited with an infrared lighting system [18]. One of the first works that employed NIR to predict the phenolic content in olive oil is detailed in [19]. In that work, the authors got a regression coefficient (*R*^2^) value of 0.89, and root mean square error in cross validation (RMSECV) of 58.669 mg/kg. Similarly, in [20] authors evaluate Partial Least Squares (PLS) models in order to assess several minor components in extra virgin olive oils. They collected NIR spectra with different spectrophotometers, and unfortunately, they reached poor correlations for total polyphenol content prediction, showing an R^2^ value of 0.34 at best. Most recently, the total phenol content prediction from near infrared spectra was studied in [21]. This work reports an *R*^2^ value of 0.85 with a PLS regression model and RMSECV of 45.10 mg/kg. To the best of our knowledge, to date, the last work that deals with NIR spectra to predict phenol content can be found in [22]. In that work, a system based on an artificial neuronal network and NIR was designed for real-time characterization of olive oil during the kneading process. The system was trained to predict polyphenol content among other parameters with an *R*^2^ of 0.89 and root mean square error of prediction (RMSEP) of 61.50 mg/kg. All referred works used olive oils with total polyphenol contents below 1000 mg/kg.

Different sources of error can disturb the measures, and the study of how they affect the calibration needs to be considered for each particular application [23]. There are different variables related to the NIR spectra acquisition setup which need to be studied and adjusted, in order to reach a reproducible and repeatable system, such as light source power and optical distance from the lens to the sample, among others. For example, the turbidity of the liquid samples can affect the measures. In olive oil, it occurs when microparticles of water and other impurities from the olive fruit go into the olive oil [24]. It increases the optical density of the liquid sample, and the power received by the NIR sensor is reduced. In this context, different works have carried out experimentations to assess the optimal parameters. Specifically, in [25], the authors evaluated the effect of some parameters, such as focal distance and integration time, on the spectral repeatability for the analysis of intact olive fruits on a conveyor belt. They employed an InGaAs diode array spectrometer, concluding that 13 was the number of the focal distance in millimeters, and 5 s was the integration time. These values gave a higher repeatability than other values. Also, [26] studied a partial least square (PLS) model to predict free acidity in olive oils. Their aim was to determine the optimal path length and spectral wavelengths. The work showed that the increase of the optical path length in data acquisition results in more reliable PLS models. Other variables, such as number of acquisitions and halogen light power, have been evaluated in this research.

A final important point is the identification of wavelengths as the features of the olive oil that are correlated with the interested compound, in order to reduce the size of the sensor and the computational time to get results. However, to our knowledge, in the literature, there are no works dealing with this issue. In any case, this is of particular relevance, as the hardware price is the factor limiting the deployment of NIR systems. 

## 3. Materials and Methods

**Figure 1:**
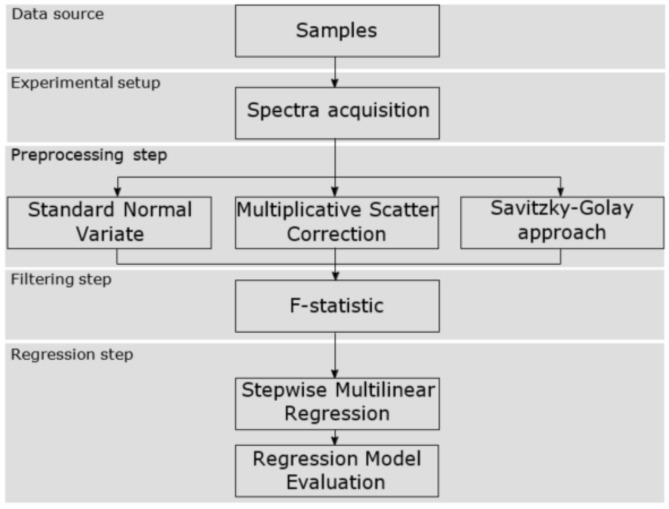
Workflow followed for our approach.

The proposed methodology that has been carried out to correlate and identify the EVOO near infrared spectra with the polyphenol content has been structured in five blocks. The first stage was to select the production process point to get the data source, i.e., the EVOO samples, so that the olive oil turbidity was in an acceptable range. Then, secondly, the spectra acquisition was carried out using the experimental setup built ad hoc. Also, as will be shown, different acquisition parameters were modified at this stage. Consecutively, in the third stage, the raw frequency values were processed with different pretreatment algorithms to remove non-desired effects in the NIR spectra. After that, in the fourth stage, the spectra were filtered in order to select certain wavelengths related to the compound of interest. Finally, the last phase consisted of building the regression model which correlates the absorbance values with the concentration of total polyphenols. Figure 1 shows the aforementioned workflow and the next sections explain the details.

### 3.1. Extra Virgin Olive Oil Samples

**Figure 2:**
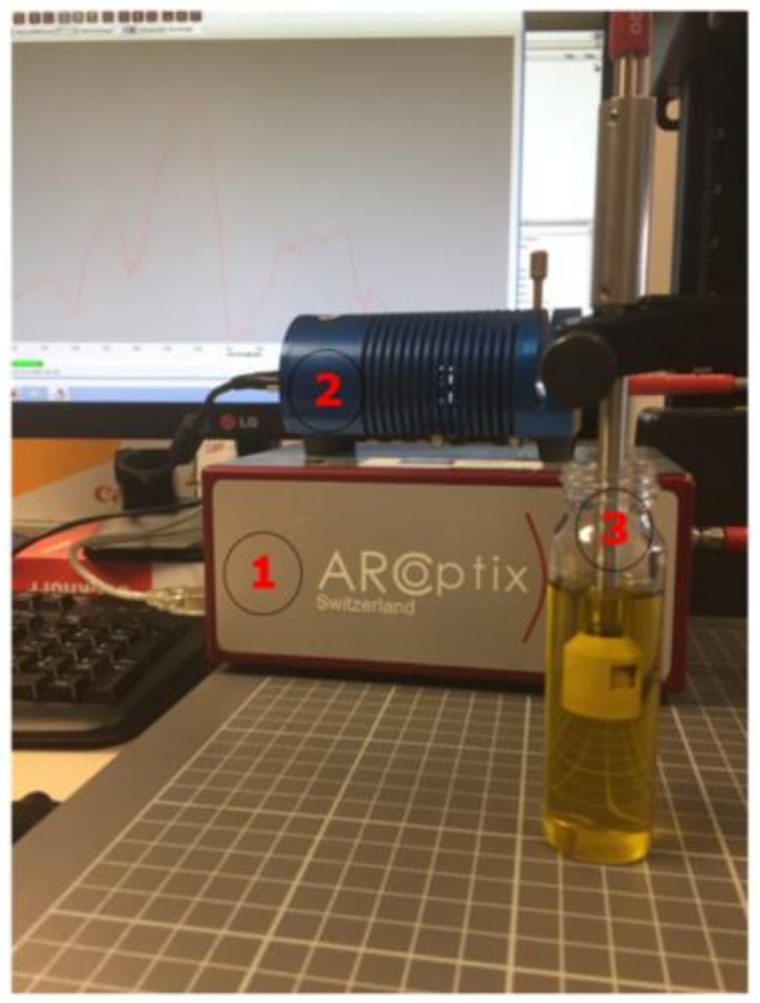
Hardware configuration labelled with numbers: (1) near infrared (NIR) sensor, (2) light source, and (3) probe.

Different olive oil batches were sampled from the olive oil factory, at the exit of the vertical centrifugal machine (Figure 2), and before the filtering step. At this point, the microdroplets of water and different solid particles have been removed from the olive oil. The samples were produced using different values of the process variables, such as malaxing temperature, malaxing time, and water addition before the oil extraction, which resulted in olive oils with different content of minor components. The initial set was composed of 11 samples with the following polyphenol concentrations in mg/kg: (1017, 1132, 1247, 1362, 1478, 1593, 1708, 1823, 1939, 2054, 2169). Finally, these samples were blended to increase the dataset to 21 olive oil samples. The blends were carried out by pouring the same quantity of olive oil from consecutive concentrations. The final dataset was composed by the following concentrations: (1017, 1074, 1132, 1189, 1247, 1305, 1362, 1420, 1478, 1535, 1593, 1650, 1708, 1766, 1823, 1881, 1939, 1996, 2054, 2112, 2169). The olive oil was monovarietal and the original olive fruit variety was Picual. On the other hand, the samples were stored at 6◦C in darkness until their chemical and spectral analysis.

This study had the collaboration of the Spanish National Research Council (CSIC) through the department of Food Biotechnology (www.ig.csic.es). They assessed the total content of polyphenols in the collected samples. Briefly, the standardized method was as follows; 0.6 g of olive oil was extracted using 3 × 0.6 mL of dimethylformamide (DMF); the extract was then washed with hexane, and N_2_ was bubbled into the DMF extract to eliminate residual hexane. Finally, the extract was filtered through a 0.22 µm pore size and injected into the chromatograph. The method is more detailed in [27].

### 3.2. Experimental Setup

The hardware employed in our experimentation can be seen in the Figure 2, which has been labelled with numbers.

It was composed by the ARCoptix Fourier transform near infrared (FT-NIR) [28] spectrometer (label number 1) which can detect the intensity of the light in the frequencies between 900 and 2600 nm, and the spectral resolution is 8 cm^−1^. The samples were radiated with the halogen light source model HL-2000-HP-232R developed by OceanOptix. Its power is 20 w in nominal bulb power, and the output can be regulated (label number 2). That lamp gives off radiation within the NIR band. The probe (label number 3) has a particular form in order to carry out measurements in transflectance mode. The devices were connected by fiber optic cable. Although the ARCoptix software was used to control the FT-NIR device, the statistical computations were carried out with Matlab R2013b (MathWorks, Natick, MA, USA).

### 3.3. Spectra Acquisition Procedure

Firstly, with the aforementioned probe, the transflectance spectra was acquired for each sample. This process was carried out in the laboratory of the Robotic, Automation and Computer Vision research group at the University of Jaén, and at room temperature (20 ± 0.5 °C). The optical path length was constant and equal to 5 mm. The transflectance methodology can be briefly explained by following the path that the light follows. Firstly, the light goes out from the halogen lamp to the sample through the optic fiber. The light goes from the tip of the probe to the sample, it traverses the oil and is reflected by a specular surface. Then, the light comes back to the optic fiber until it arrives to the near infrared sensor.

The number of captures was 10 for each sample; however, each acquisition was saved independently. Then the transflectance spectra were coded in matrix form (Equation (1)) where rows (I) are the sample number and columns (Υ) are the light power transmitted by the sample for each wavelength. In our case, I was 21 and Υ was equal to 921.

X = (x_iυ_,i = 1, 2, ..., I; υ = 1, 2, ..., Υ) (1)

The power received (P_r_) by the NIR device follows the classical energy balance and it depends on the transmitted power by the halogen lamp (P_t_), the power light that has been absorbed by the olive oil samples (P_abs_) and different losses caused by hardware components, such as connectors or multiplexors in a multipoint system (L). The energy balance equation for each wavelength (υ) is treated in Equation (2).

P_r_(υ) = P_t_(υ) − P_abs_(υ) − L(υ) (2)

**Figure 3:**
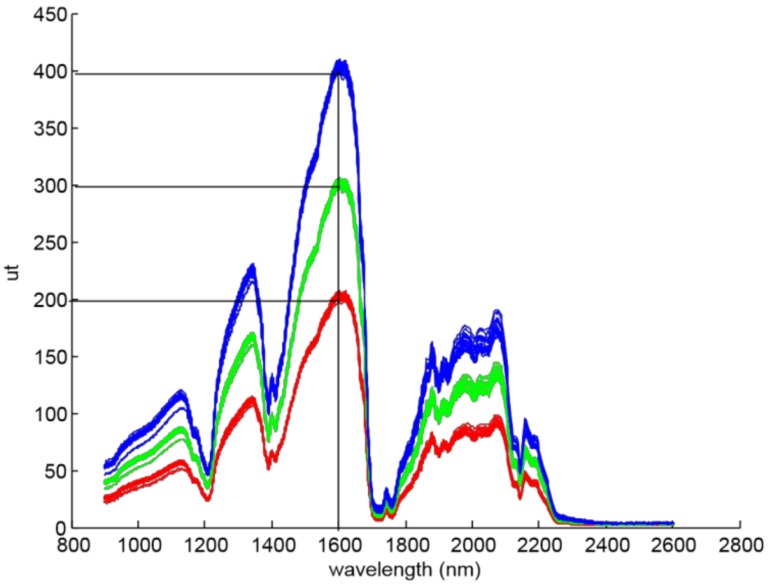
Olive oil sample spectra for the three groups: S1 in red, S2 in green, and S3 in blue.

One of the aims of this work is to present the effect of the halogen light power on prediction results. Therefore, three P_r_(υ) targets were fixed for each acquisition, 200 ut, 300 ut and 400 ut, where ut are the units of transflectance, and show the raw value obtained by the analogue to digital converter of the NIR device. Also, the referenced wavelength υ was set in 1600 nm, because it is the maximum value of olive oil transflectance spectrum (Figure 3). Then, three groups of spectra (S1, S2, and S3) were built, and the output light power was adjusted for each group in order to reach the targeted Pr(1600 nm) value (Table 1).

### 3.4. Spectra Preprocessing

Firstly, the raw spectra were transformed from transflectance values to absorbance values. The spectra of absorbance show the quantity of light that is absorbed at different wavelengths. It is useful when the purpose is to build a calibration model [29].

(3) $$X_{abs}\left(\upsilon \right)=log_{10}\left(\frac{1}{X_{trans}\left(\upsilon \right)}\right)$$

Therefore, Equation (3) was applied to the whole dataset, where *X_abs_* are the absorbance spectra and *X_trans_* are the transflectance spectra. The NIR sensor sensitivity is not the same among the different wavelengths, and there are spectral bands where the signal-to-noise ratio is poor due to the low gain (see Figure 3, from 2300 nm to 2600 nm).

(4) $$CV\left(\upsilon \right)=\frac{s\left(\upsilon \right)}{\bar{x}\left(\upsilon \right)}\times 100$$

Thus, the coefficient of variation (*CV*) (Equation (4)) was applied for each sample and each wavelength, in order to detect these spectral bands, where *s* is the sample standard deviation and $\bar{x}$ is the sample mean for each wavelength υ.

Furthermore, the acquired spectra were preprocessed to remove and/or correct some disturbances in the measurements. The disturbances could be due to scattering effects in the olive oil according to microparticles or inhomogeneities of the surface. Also, they can be due to differences in the spectrum baseline based on differences in the optical path length or interferences from external light and random noise. Normally, the success ratio of the preprocessing algorithms can be evaluated by experimental tests. Firstly, the classification or regression model is configured, and then the error of the model is computed with the same inputs, but preprocessed with different algorithms. The pre-treatment methods studied in this work were the standard normal variate (*SNV*) [30], multiplicative scatter correction (MSC) [31], and Savitzky–Golay (SG) [32] derivative method.

(5) $$x_{i\upsilon ,SNV}=\frac{\left(x_{i\upsilon }-{\bar{x}}_{i}\right)}{\sqrt{\frac{\sum_{\upsilon =1}^{\Upsilon }{\left(x_{i\upsilon }-{\bar{x}}_{i}\right)}^{2}}{\Upsilon -1}}}$$

The *SNV* is a pretreatment method used quite often in NIR to remove the scatter added to the spectrum. Its approach is to calculate the average and standard deviation of all the data points for that spectra, and each data point of the spectra is subtracted from the mean and divided by the standard deviation. It is applied to all the spectrum individually, and it allows one to compare different spectra on the basis of the same reference (Equation (5)), where *x_iυ,SNV_* is the transformed element for original element x_iυ_, and ${\bar{x}}_{i}$ is the mean of spectrum *i*, and Υ is the number of variables in the spectrum.

On the other hand, the MSC methodology is normally used to detect additive and/or multiplicative effects spectral signals. It detects and removes physical effects, like heterogeneities in the size of particles, and corrects the intensity of the wavelengths in the spectrum which do not carry any chemical or physical information.

(6) $$x_{i}=a_{i}+b_{i}\bar{x_{\upsilon }}+e_{i}$$

Firstly, each spectrum is fitted to the average spectrum by least squares (Equation (6)), where *x_i_* is the spectrum of an individual sample *i*, $\bar{x_{\upsilon }}$ is the mean spectrum of the group, and the error *e_i_* corresponds to all other effects in the spectrum that cannot be modelled by an additive and multiplicative constant, in other words, it represents the chemical differences among the olive oil samples.

(7) $$x_{i,MSC}=\frac{x_{i}-a_{i}}{b_{i}}$$

The corrected spectrum *x_i,MSC_* is calculated using the fitted constants *a_i_* (intercept) and *b_i_* (slope) with the Equation (7).

Alternatively, the derivative approach, named Savitzky–Golay method [32], was evaluated in order to remove possible overlapping peaks and correct the spectra baseline. It is a spectral low-pass filtering method where a convolution is used instead of the mean of the spectrum.

(8) $$x_{i\upsilon ,SG}=\frac{\sum_{n=-N/2}^{N/2}C_{n}x_{i,\upsilon +n,}}{N}$$

Its mathematical expression appears in Equation (8), where *C_n_* is the convolution coefficient for the spectral value *υ*, and *N* is the size of the window. The convolution coefficients are obtained using minimum squares adjustment of the points of the spectrum to a polynomial of determined grade.

The result is a spectrum similar to the input one, but smoother. This approach saves the features of the original signal, such as maximums, minimums, widths of the peaks, and preserving the original distribution. Also, it is useful to remove, at the same time, the baseline of the spectra, if it is applied in derivative form. 

### 3.5. Spectra Filtering

The amplitude of the absorption at any particular wavelength is determined by its absorptivity and the number of molecules encountered within the beam path of the measuring instrument. This relationship is described by Beer’s law [33]. Then, the absorptivity is not the same throughout the spectrum, and there are wavelengths that provide more information than others related to the studied compounds.

In this step, the one-way ANOVA analysis was employed in order to compare the features between samples in the same class and samples in different classes. It was performed by defining two classes: the first class was composed by samples with high concentrations of polyphenols, and the second class with samples with low concentrations. The aim was to remove irrelevant wavelengths from the feature vector, and search the most discriminant wavelengths. If the ratio of within-group variation to between-group variation for one wavelength is significantly high, we can conclude that the group means are significantly different from each other. We can measure this using a test statistic that has an *F*-distribution with (k − 1, N − k) degrees of freedom. This test was applied for each wavelength. If the *p*-value for the *F*-statistic is smaller than the significance level, then the test rejects the null hypothesis that all group means are equal, and concludes that at least one of the group means is different from the others. The most common significance levels are 0.05 and 0.01. In our case, features with *p*-value lower than 0.05 were candidates to be removed.

### 3.6. Dimensionality Reduction with Regression Models

There are two general approaches for performing dimensionality reduction: feature selection models and feature extraction models [34]. The first one identifies a subset of features without applying any transformations, and the second one employs mathematical operations in order to transform the originals features into a lower dimensional space. The algorithm evaluated in this work was the stepwise multilinear regression (SMLR) [35] algorithm, in order to evaluate the effect in the prediction results when different wavelengths were introduced in the model. It uses the polyphenol content as dependent variable (Y), and wavelengths as independent variables (X).

Stepwise multilinear regression model is an iterative algorithm, and it consists on adding and removing terms from a linear model based on their statistical significance in explaining the response value. The method begins with an initial model, and then compares the explanatory power of incrementally larger or smaller models.

SMLR uses forward and backward stepwise regression to build the final model. At each step, the algorithm searches for wavelengths to add or remove from the model according to a specific criterion. In our case, the criterion was to use the statistical *p*-value and *F*-value to test models with and without a potential wavelength at each step. Then, if a wavelength is not currently in the model, the null hypothesis (H_0_) is that the coefficient attached to the wavelength would have a zero value if it is added to the model (forward approach). If the null hypothesis is rejected, the wavelength is added to the model. Furthermore, if a wavelength is currently in the model, the H0 is that the coefficient of the wavelength is equal to zero. If there is insufficient evidence to reject the null hypothesis, the wavelength is removed from the model (backward approach) [36].

The algorithm was applied throughout the next steps:

- Step 1: Fit a linear model with β_0_ and the set of selected (B) wavelengths:
  Y = β_0_ + X × B (9)
- Step 2: For β*_k_* (*k* from 1 to number of wavelengths) not in B iterate from 3 to 6.
- Step 3: Adjust the lineal model:
  (10) $$y_{n}=\beta_{0}+\underset{B}{\sum }x_{n,k}\beta_{k}$$
- Step 4: Null hypothesis: H_0_: β*_k_* = 0
- Step 5: *F*-value and *p*-value are obtained.
- Step 6: If *p*-value < 0.05: β*_k_* is added to the set B and go to Step 1.

Finally, the regression model was evaluated with leave one out cross validation (LOOCV) and holdout validation. LOOCV is an iterative validation wherein one sample *x_i_* is removed from the dataset in each iteration. The number of iterations is equal to the number of samples (*N*). Then the model is built with the rest of samples in the dataset, and it predicts the *y_i_* related to the previously removed sample *x_i_*. On the other hand, holdout validation employs part of the dataset for training, and the rest of samples to validation purpose. In our case, the criterion was 50%/50% and the samples in each group were selected randomly.

(11) $$RMSE=\sqrt{\frac{\sum_{i=1}^{N}{\left(y_{i}-\hat{y_{i}}\right)}^{2}}{N}}$$

(12) $$R^{2}=1-\frac{\sum_{i=1}^{N}{\left(y_{i}-\hat{y_{i}}\right)}^{2}}{\sum_{i=1}^{N}{\left(y_{i}-\bar{y_{i}}\right)}^{2}}$$

The predicted value is represented as $\hat{y_{i}}$. At the end, the root mean square error (RMSE) is obtained according to the Equation (11). Furthermore, the determination coefficient *R*^2^ is calculated in order to evaluate how the variability of the response variable Y is explained by the regressor variable X. It is explained with the Equation (12).

## 4. Results and Discussion

**Figure 4:**
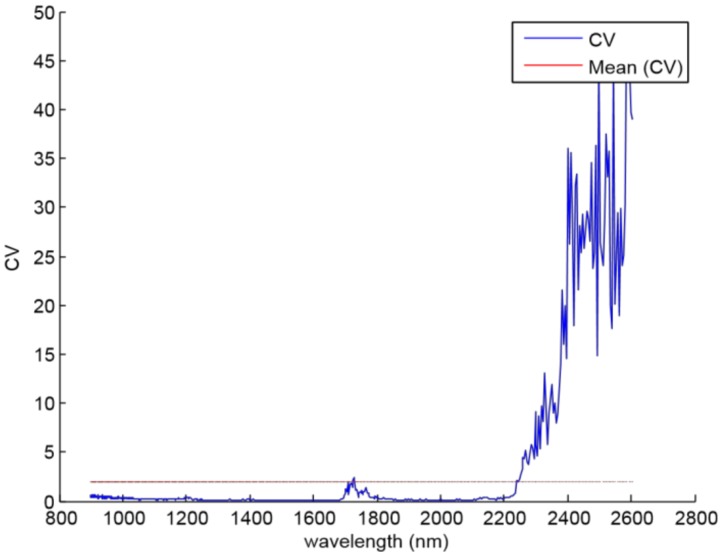
Blue line represents the CV coefficients for each wavelength; red line is the CV mean value.

The noisy spectral bands of the NIR sensor were detected by applying the CV coefficient to the whole wavelengths. The criterion was to remove the components whose CV value was greater than its mean value. Then, Figure 4 shows that the noisy spectral bands are located between 2240 and 2600 nm, and it is due to the loss of power caused by the experimental setup.

**Figure 5:**
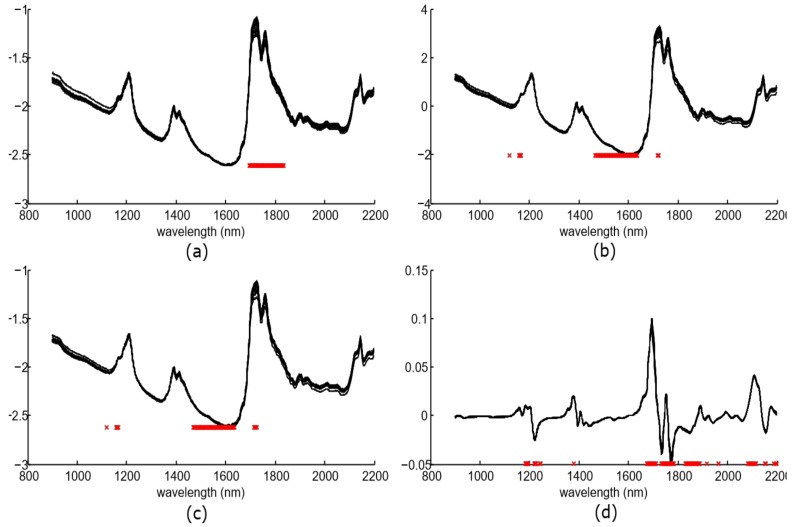
Figure shows the selected wavelengths after the filtering step (red marks) and the spectra of the whole dataset for each preprocessing algorithm: (**a**) without preprocessing, (**b**) standard normal variate, (**c**) multivariate scatter correction and (**d**) Savitzky–Golay approach.

Spectra were preprocessed with the aforementioned algorithms, and the *F*-statistic and the *p*-value were obtained for each wavelength. Significant wavelengths (with *p*-value lower than 0.05) were selected (up to a limit of 100 components) while the rest were removed from the feature vector. After that, the wavelengths were arranged in descending order according to their *F*-statistic values. Figure 5 shows the preprocessing spectra for the whole dataset and the chosen components.

The number of these significant components was 57 for spectra without preprocessing, 100 for SNV, 100 for MSC, and 98 for the SG approach. The majority of these components were located at the spectral bands of 1185–1245 nm, and 1673–1965 nm. This spectral bands concur with other works such as [21,37], where authors found relations with the vibrations the OH bond presents in the hydroxyl molecular group.

SMLR model was applied with the selected wavelengths, and the experimental results were evaluated on the basis of the prediction error. Table 2 shows the calibration and validation performance in terms of *RMSE* and *R*^2^ for each preprocessing algorithm. 

**Figure 6:**
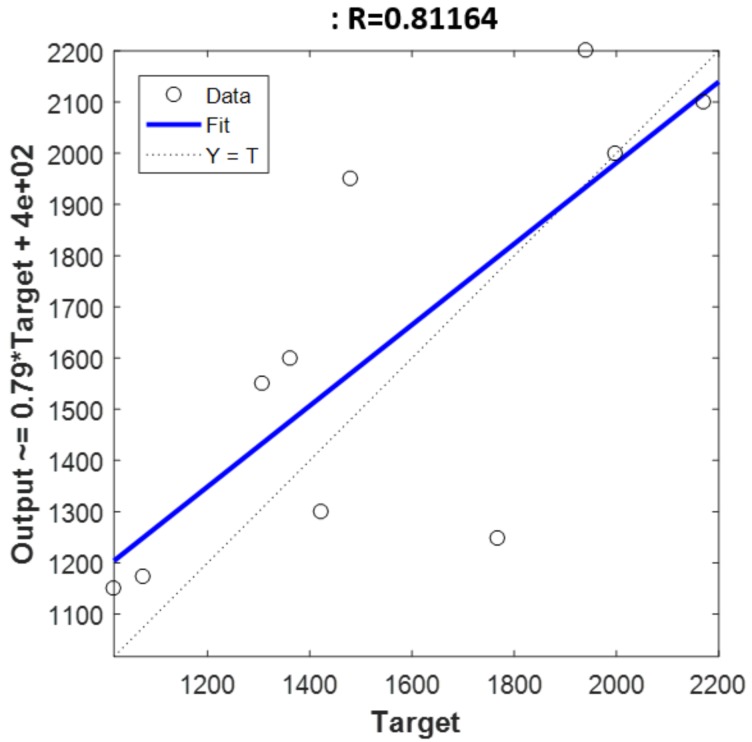
Figure shows the correlation between the observed (x-axis) vs. the predicted values (y-axis) for holdout validation.

The best *R*_v_^2^ value was reached when the holdout validation was applied to the dataset. It reached a value of 0.81 while RMSEV value was 255.9 mg/kg. Figure 6 shows the predicted values by the model versus the original values obtained through chemical analysis. On the other hand, the best RMSEV result was reached by SMLR method with SG approach, obtaining a *R*_v_^2^ coefficient of 0.80 and a RMSEV value of 156.14 mg/kg, that is 13.55% over the reference range of polyphenol content. The studies developed in [22] reached an error value of 61.50 mg/kg with PLS regression model, however, they employed a different reference range of polyphenol concentrations below 1000 mg/kg. Also in [19], the results were similar, and the authors pointed out that better calibration could be achieved with a wider distribution. In our work, the linear model that defines the best correlation has three components.

(13) $$\hat{y}=\beta_{0}+\beta_{1}x_{11}+\beta_{2}x_{38}+\beta_{3}x_{72}$$

That model appears in Equation (13), where $\hat{y}$ is the predicted value, $x_{11}$, $x_{38}$, and $x_{72}$ are the absorbance values at 1830 nm, 1917 nm, and 2189 nm, respectively, and $\beta_{0}$, $\beta_{1}$, $\beta_{2}$, and $\beta_{3}$ are the estimated regression coefficients, and their values are −5453, −4.16 × 10^5^, 1.56 × 10^5^, and 1.92 × 10^5^, accordingly.

**Figure 7:**
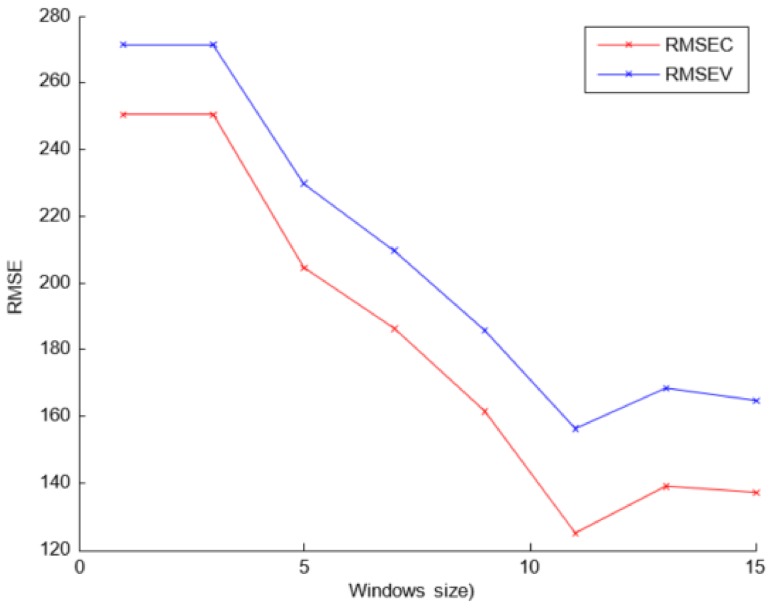
The RMSEC and RMSEV values when different set of samples were employed and different number of acquisitions per sample were averaged.

On the other hand, the preprocessing SG algorithm was employed by applying the first derivative to the spectra, with a fitting polynomial of second degree and a window size of 11 points. The last parameter was selected experimentally, and Figure 7 presents the RMSE results for each window size. 

**Figure 8:**
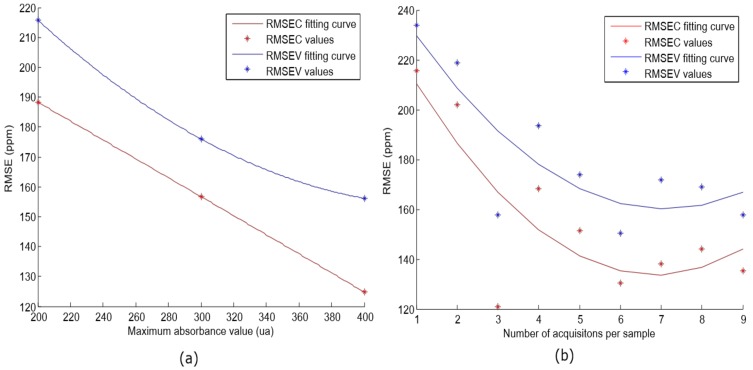
(**a**,**b**) show the RMSEC and RMSEV values when different sets of samples were employed and different numbers of acquisitions per sample were averaged, respectively.

It suggests that the window size of 11 components achieves the best RMSEC and RMSEV results. Finally, the lineal model was evaluated with different acquisition parameters (maximum absorbance value and number of acquisitions per sample). As indicated in the previous section, different sets of samples were built (S1, S2, and S3). The same SMLR algorithm was applied for each set, and the results are supplied in Figure 8a. 

This shows a decreasing trend in RMSE value according to the increase of the maximum absorbance value. A similar study was carried out in [26], where authors evaluated different optical path lengths for free acidity determination, and they concluded that lower path length seemed to work better. The results were the same, since the received power to the detector is inversely related to the path length. However, in our case, it depends on the maximum output power of the halogen lamp as a restriction. Furthermore, the number of acquisitions per sample was modified (from 1 to 10) in order to evaluate how it affects the performance. The S3 set was employed for this experiment, and Figure 8b shows the results. Since the spectra is averaged, the noise is reduced. Up to 6 acquisitions occur where the minimum RMSEC and RMSEV values are reached. Similar results were reached in [25], where authors concluded that results were improved when the integration time for their NIR device was higher. For our case, the integration time can be related to the number of acquisitions per sample. These results are useful to adjust the optimal number of acquisitions and the halogen lamp features in order to adjust and import the setup in the olive oil elaboration process.

## 5. Conclusions

This work describes a methodology to obtain and improve the correlations between olive oil NIR spectra and polyphenol content. The best correlated wavelengths were identified, and two acquisition parameters were modified in order to reduce the cross-validation error. The proposed approach was based on three stages: (1) selection of samples and spectra acquisition with the experimental setup; (2) preprocessing step and filtering step; and (3) regression model implementation and validation. Different preprocessing algorithms were evaluated, such as standard normal variate, multivariate scatter correction and Savitzky–Golay method. The preprocessed spectra were filtered by selecting the significant components, and a specific regression algorithm was employed based on feature selection approach.

The results showed the existence of correlations between olive oil NIR spectra and polyphenol content by using the proposed methodology. The best results were reached with the lineal model obtained by the SMLR regression algorithm with Savitzky–Golay preprocessing algorithm. It got a RMSEV value of 156.14 mg/kg with an *R*_v_^2^ value of 0.80 when leave one out cross validation was applied. When holdout validation was applied, the *R*_v_^2^ value was nearly the same (0.81), while RMSEV was worse (255.9 mg/kg). Furthermore, the significant wavelengths included on the linear model were 1830 nm, 1971 nm, and 2189 nm. In the view of the results, the proposed approach could be useful for qualitative determination in the industrial factory rather than quantitative purposes.

Finally, the linear regression model was applied with different acquisition parameters, such as halogen lamp power and number of acquisition per sample. These parameters need to be taken into consideration in order to design an industrial system. The model performance improved lineally with the increase of the halogen lamp output power. Also, the optimal number of acquisitions to average was 6 for our purposes, where RMSEV decreased to 150.23 mg/kg. In future work, other factors could be taken into consideration, such as olive oil temperature or moisture content.

## Figures and Tables

**Table 1:** This table shows the number of samples for each studied group (S1, S2, and S3), the maximum value for each group and their standard deviation.

Set Number	Number of Samples	Target Value	Reached Value	Standard Deviation
S1	21	200	202.39	2.53
S2	21	300	301.74	2.37
S3	21	400	403.75	3.38

**Table 2:** Experimental results.

Regression Algorithm	Preprocessing Algorithm	Validation	Number of Components after Filtering	Number of Components after Regression	RMSEC (mg/kg)	*R*_c_^2^	RMSEV (mg/kg)	*R*_v_^2^
SMLR	ABS	LOOCV	57	3	155.05	0.80	188.29	0.70
SMLR	ABS+SNV	LOOCV	100	1	191.56	0.69	211.25	0.63
SMLR	ABS+MSC	LOOCV	100	1	189.62	0.70	208.23	0.64
SMLR	ABS+SG	LOOCV	98	3	124.78	0.87	156.14	0.80
SMLR	ABS	HOLDOUT	57	3	155.05	0.80	291.92	0.62
SMLR	ABS+SNV	HOLDOUT	100	1	191.56	0.69	493.63	0.70
SMLR	ABS+MSC	HOLDOUT	100	1	189.62	0.70	243.03	0.73
SMLR	ABS+SG	HOLDOUT	98	3	124.78	0.87	255.90	0.81
